# Late onset toxicities associated with the use of CDK 4/6 inhibitors in hormone receptor positive (HR+), human epidermal growth factor receptor-2 negative (HER2-) metastatic breast cancer patients: a multidisciplinary, pan-EU position paper regarding their optimal management. The GIOCONDA project

**DOI:** 10.3389/fonc.2023.1247270

**Published:** 2023-10-26

**Authors:** Marina Elena Cazzaniga, Antonio Ciaccio, Romano Danesi, Francois P. Duhoux, Corrado Girmenia, Kalhil Zaman, Henrik Lindman, Fabrizio Luppi, Dimitrios Mavroudis, Ida Paris, Ayodele Olubukola, Ahmed Samreen, Christian Schem, Christian Singer, Anton Snegovoy

**Affiliations:** ^1^ Phase 1 Research Unit, IRCCS San Gerardo dei Tintori, Monza (MB), Italy; ^2^ School of Medicine and Surgery, Milano Bicocca University, Monza (MB), Italy; ^3^ Gastroenterologic Unit, IRCCS San Gerardo dei Tintori, Monza (MB), Italy; ^4^ Department Experimental and Clinical Medicine, University of Pisa, Pisa, Italy; ^5^ Cliniques Universitaires Saint-Luc, UC Louvain, Brussels, Belgium; ^6^ Department of Hematology, Oncology and Dermatology, Azienda Ospedaliero-Universitaria Policlinico Umberto I, Roma, Italy; ^7^ Breast Center, Department Of Oncology, Lausanne University Hospital CHUV, Lausanne, Switzerland; ^8^ Department Of Immunology, Genetics And Pathology, Uppsala University, Uppsala, Sweden; ^9^ Division Of Respiratory Diseases, Fondazione IRCCS San Gerardo Dei Tintori, Monza (MB), Italy; ^10^ Department Of Medical Oncology, University Hospital Of Heraklion, Crete, Greece; ^11^ Department of Woman and Child Health and Public Health, Fondazione Policlinico Universitario Agostino Gemelli IRCCS, Roma, Italy; ^12^ University Hospitals of Leicester NHS Trust, Leicester, United Kingdom; ^13^ University Hospitals Of Leicester Osborne Building, Leicester Royal Infirmary, Leicester, United Kingdom; ^14^ MaHM Mammazentrum, Hamburg, Germany; ^15^ Department Of Obstetrics & Gynecology Head, Center For Breast Health, Medical University of Vienna, Vienna, Austria; ^16^ Department Of Oncology, University Of Medicine & Dentistry, Moscow, Russia

**Keywords:** CDK 4/6 inhibitors, abemaciclib, ribociclib, palbociclib, diarrhea, liver toxicity and injury, interstitial lung disease

## Abstract

The personalization of therapies in breast cancer has favoured the introduction of new molecular-targeted therapies into clinical practice. Among them, cyclin-dependent kinases 4 and 6 (CDK4/6) inhibitors have acquired increasing importance, with the approval in recent years of palbociclib, ribociclib, and abemaciclib in combination with endocrine therapy. Currently, no guidelines are available to monitor and manage potential long-term toxicities associated with the use of these drugs. A multidisciplinary panel of European oncologists, was supported by a pharmacologist, a hematologist, a hepatologist and a pulmonologist to discuss the management of long-term toxicities, based on the literature review and their clinical experience. The panel provided detailed roadmaps to manage long-term toxicities associated with the use of CDK4/6 inhibitors in clinical practice. Knowing the frequency and characteristics of the toxicity profile associated with each CDK4/6 inhibitor is important in the decision-making process to match the right drug to the right patient.

## 1 Introduction

The personalization of therapies in breast cancer has favoured the introduction of new molecular-targeted therapies into clinical practice. Among them, cyclin-dependent kinases 4 and 6 (CDK4/6) inhibitors have acquired increasing importance, with the approval in recent years of palbociclib, ribociclib, and abemaciclib in combination with endocrine therapy (ET) (1). The use of CDK4/6 inhibitor therapy has gained ground since its approval in Europe in late 2016 (2) and the latest European Society for Medical Oncology (ESMO) consensus guidelines for advanced breast cancer describe a CDK4/6 inhibitor combined with endocrine therapy as “the standard-of-care first-line therapy” for patients with HR+/HER2 − metastatic breast cancer (3). Randomized clinical trial data indicated that the use of ribociclib improved the overall survival (OS) in both pre-/perimenopausal patients [58.7 months with ribociclib versus 48.0 months with placebo; hazard ratio = 0.76; 95% confidence interval (CI), 0.61-0.96] (4) and postmenopausal patients with a median OS of 53.7 months versus 41.5 months with placebo [hazard ratio (HR), 0.73; 95% confidence interval (CI) 0.59-0.90] (5).

Even, palbociclib plus aromatase inhibitors (AI) improved both PFS and, in a real-world setting, also the OS compared to AIs alone (6). Similarly, real-world experience with abemaciclib confirmed the clinical benefit observed in trials, although patients showed characteristics typically indicative of a less favorable prognosis in the former setting (7).

Although these drugs share a similar molecular mechanism of action and metabolism by the CYP3A4, they differ substantially in terms of half-life, IC_50_, lipophilicity, and their binding avidity to different CDK molecules (8).

These differences contribute to the disparity in their toxicity profiles. Overall, the three CKD4/6 inhibitors are comparable in terms of any grade toxicities, with abemaciclib showing a lower risk of grade 3-4 toxicities, with an absolute risk (AR) of 0.592 (95% confidence interval -CI- 0.557-0.626; p< 0.0001) compared to palbociclib (AR 0.763, 95% CI 0.634-0.857; p < 0.0001) and ribociclib (AR 0.739, 95% CI 0.629-0.825; p < 0.0001) as resulted in a meta-analysis of both randomized clinical trials and observational studies (9).

Knowing the frequency and characteristics of the toxicity profile associated with each CDK4/6 inhibitor is important in the decision-making process to match the right drug to the right patient.

Currently, no guidelines are available to monitor and manage potential long-term toxicities associated with the use of CDK4/6 inhibitors. Therefore, in this project, a multidisciplinary panel of experts collected and commented on data regarding the long-term toxicities of CDK4/6 inhibitors, especially those with late-onset during the treatment and/or under-reported or not collected in clinical trials. In addition, the panel discussed the results of a survey that specifically explored the attitudes toward managing such toxicities in everyday practice across European centers. The final goal was to propose a position paper on practical suggestions for the optimal management of late toxicities related to these targeted agents, based on both literature evidence and expert opinion.

## 2 Assessment of policy/guidelines options and implications

A multidisciplinary panel of European clinicians from Italy, Belgium, Sweden, Switzerland, Greece, Austria, Germany, the United Kingdom, and Russia, convened at the meetings organized within the GIOCONDA project held online in January and June 2022. The panel included eleven oncologists, a pharmacologist, a hematologist, a gastroenterologist, and a pulmonologist.

A literature search for hepatic, hematological, and pulmonary toxicities was performed in Pubmed/Medline to collect the most relevant evidence concerning these topics, and data from real-life and case reports were carefully analyzed. In parallel with the literature search, a survey was sent to groups of oncologists in 9 European countries to collect information on the management of toxicities associated with CDK4/6 inhibitors in their clinical practice (**Supplementary Table 1**).

Online meetings were held to promote a discussion between the panel of experts on available literature evidence and clinical practice attitudes that emerged from the survey results.

### 2.1 Literature review

As the most common toxicities are hematologic for palbociclib and ribociclib and gastrointestinal for abemaciclib (9), the literature review focussed on these adverse reactions. An insight into pulmonary, cardiac, and renal toxicities was also provided.

#### 2.1.1 Hematologic toxicity

The most common grade 3-4 hematologic toxicity during CDK4/6 inhibitor treatments is neutropenia while other hematologic toxicities (i.e., anemia and thrombocytopenia) are uncommon and of mild to moderate severity.

The results of a meta-analysis by Onesti et al. indicated that the absolute risk of neutropenia of any grade and grade 3-4 was higher with palbociclib and ribociclib compared to abemaciclib; febrile neutropenia was observed at a higher rate with palbociclib (AR 0.023, 95% CI 0.017-0.031, p < 0.0001) than with ribociclib (AR 0.010, 95% CI 0.005-0.021, p< 0.0001) and abemaciclib (AR 0.008, 95% CI 0.002-0.032, p < 0.0001) (9). Lower odds of grade 3–4 neutropenia between abemaciclib and palbociclib, when used with either aromatase inhibitors (odd ratio OR: 0.14, p = 0.03) or fulvestrant (OR: 0.06, p = 0.01) were reported (10). Ribociclib showed a more favorable hematologic toxicity profile than palbociclib, with less grade 3–4 neutropenia (OR: 0.39–0.41 depending on endocrine therapy backbone) and anaemia (OR: 0.45–0.79 depending on endocrine therapy backbone) (10).

Nonetheless, despite the elevated absolute risk of grade 3-4 neutropenia, febrile neutropenia, and severe infections occur in less than 5% of patients and, regardless of the severity of neutropenia, the infectious risk is much lower during neutropenia secondary to CDK4/6 inhibitors than post-chemotherapy. Indeed, biological mechanisms responsible for neutropenia caused by CDK4/6 inhibitors versus chemotherapy are different. Unlike chemotherapy, *in vitro* CDK4/6 inhibitors cause cell cycle arrest but no death of proliferating neutrophil precursor cells, thus allowing for a rapid and spontaneous recovery of the neutrophil count after treatment discontinuation (11). For these reasons, palbociclib and ribociclib are administered for three consecutive weeks followed by a week’s break, to allow recovery of hematopoietic progenitors. Conversely, abemaciclib can be administered continuously, since it shows a lower rate of hematopoietic toxicity than either palbociclib or ribociclib. Although the rate of infections is low, an increased risk of infections of all grades (HR 1.77, 95% CI 1.56-2.01, p < 0.00001), of grade 3 or higher (HR 1.77, 95% CI 1.28-2.43, p = 0.0005), and urinary tract infections (HR 1.59, 95% CI 1.19-2.12, p = 0.002) in patients treated with CDK4/6 inhibitors and closer follow-up for infections in these patients could prevent complications and early death (12).

Granulocyte colony-stimulating factor (G-CSF) is generally not required for the management of CDK4/6 inhibitor-induced neutropenia, while a rapid neutropenia recovery is obtained by dose interruption and dose modification of the CDK4/6 inhibitor without modifying the endocrine agents.

In presence of unusual and persistent neutropenia, anemia, and thrombocytopenia a metastatic bone marrow infiltration should be suspected and investigated.

#### 2.1.2 Gastrointestinal toxicities

The most common gastrointestinal toxicity was diarrhea, with a higher absolute risk with abemaciclib, both for any grade toxicity and grade 3-4.

Compared to palbociclib, abemaciclib had approximately 4-fold higher odds for lower grade toxicity (OR: 3.62, p < 0.001 with aromatase inhibitors and OR: 4.28, p < 0.001 with fulvestrant) and at least five times higher odds for grade 3-4 diarrhea (OR: 5.04, p = 0.09 with AI and OR: 755.97, p < 0.001 with fulvestrant) (10). In the meta-analysis by Onesti et al, the absolute risk of diarrhea of any grade was 0.144, 0.258, and 0.853 for palbociclib, ribociclib, and abemaciclib, respectively (9). In the same analysis, grade 3-4 diarrhea was very infrequent for palbociclib and ribociclib, while an AR as high as 0.135 was found for abemaciclib (9). A detailed analysis of the MONARCH plus study revealed that diarrhea was early in onset (more than 65% of events presented at the first cycle, and usually at one week from treatment start), mild to moderate in grade, with less than 5% of grade 3 diarrhea reported and no grade 4 in this study (13). The duration of this event was limited to 2-4 days in those with grade 3 diarrhea; no data about the duration and recurrence of grade 1-2 diarrhea were provided, but the authors reported 1129 episodes for 246 patients experiencing diarrhea. Similarly, the analysis of MONARCH 2 and 3 trial data reported a median duration of diarrhea between 9 and 12 days for grade 2 events and between 6 and 8 days for grade 3, the latter with an incidence of 10-13% (14). Among other GI symptoms, nausea (43%), vomiting (27%), and abdominal pain (33%) were reported in abemaciclib trials with a significantly higher incidence than for other CDK4/6 inhibitors (9, 14).

#### 2.1.3 Hepatic toxicities

CDK4/6 inhibitors have chemical and pharmacokinetic features that may result in some predictable liver toxicity: their metabolism is driven by liver cytochrome CYP3A4, and the excretion of metabolites is via biliary flow. Liver-related events associated with the use of CDK4/6 inhibitors were reported from initial data of pivotal trials and, then, in real-world (**Supplementary Table 2**).

In a meta-analysis of nine phase 3, randomized clinical trials (RCT) accounting for a total of 5809 patients in the treatment arm and 4638 patients in the control arm, the incidence of ALT elevation of any grade was 13% among treated patients and 5.3% among controls (relative risk RR 2.18). Incidence of grade 3-4 liver toxicity was 4.1% *vs.* 0.8% (RR 4.4), and no fatal cases were reported (15). Onesti et al. showed detailed toxicity incidences for each CDK4/6 inhibitor: absolute risk for grade 3–4 ALT increase was 0.034 for palbociclib, 0.097 for ribociclib, and 0.046 for abemaciclib; a similar absolute risk was found for grade 3-4 AST increase (0.029, 0.054, and 0.029 for palbociclib, ribociclib, and abemaciclib, respectively) (9).

Liver injury deriving from CDK4/6 inhibitors, namely from ribociclib, has usually a cytolytic pattern -in which the ratio between normalized ALT and normalized ALP is greater than 5- with onset after a median 16 weeks of therapy (range 2-135) and recovery since CDK4/6 interruption after a median 43 days (16). Numerous very severe hepatotoxicities which meet Hy’s law definition (AST and/or ALT elevation more than 3 x ULN and a bilirubin level above 2 x ULN in the absence of cholestasis) are described in the literature, mainly for ribociclib (17). In a small proportion of cases of CDK4/6 inhibitors-induced liver damage, a liver biopsy was performed showing features of immune-mediated hepatitis that potentially explain the clear efficacy of corticosteroids in some case reports (18).

Switching to another CDK4/6 inhibitor is a valuable option to avoid long off-therapy intervals, and published reports show a very good liver safety profile even in patients who have experienced grade 3-4 liver injury from the first CDK4/6 inhibitors prescribed (19). Lastly, some evidence indicated a link between steatosis and hypertriglyceridemia and an increased risk of ribociclib-induced hepatotoxicity (20).

#### 2.1.4 Pulmonary and cardiac toxicities

The occurrence of pneumonitis was first described in clinical trials and case reports, which included also fatal cases of pneumonia in patients treated with CDK4/6 inhibitors (**Supplementary Table 3**).

Recently, Zhang et al. published a systematic review and meta-analysis evaluating the overall incidence and risk of ILD/pneumonitis related to CDK4/6 inhibitors in RCTs. In 12 RCTs, a total of 16,060 patients were eligible. The overall incidence of all-grade ILD/pneumonitis was 1.6% (131/8407) in the treatment group compared with 0.7% (50/7349) in the control group, thus suggesting that CDK4/6 inhibitors significantly increased the risk of all-grade ILD/pneumonitis; a higher incidence of grade 3 or higher ILD/pneumonitis was observed in the treatment group (21). In another meta-analysis, ribociclib exhibited a higher absolute risk compared to palbociclib for respiratory toxicity (AR for any grade respiratory toxicity 0.311 and 0.144; AR for grade 3-4 respiratory toxicity 0.020 for ribociclib and 0.012 for palbociclib) and QTc prolongation (AR for any grade QTc prolongation 0.073 and 0.008; AR for grade 3-4 QTc prolongation 0.019 and 0.002 for ribociclib and palbociclib, respectively). Insufficient data were available for abemaciclib to perform the meta-analysis (9).

Furthermore, in an observational, retrospective pharmacovigilance analysis of the FDA Adverse Event Reporting System database, Raschi and colleagues performed the first large-scale post-marketing safety study, investigating the occurrence of ILD in these patients (22), and confirmed that ILD reports represented 2.1% and 0.3% of all reports for abemaciclib and palbociclib/ribociclib, respectively. ILD occurred at recommended daily doses, with median latency ranging from 50 (abemaciclib) to 253 (ribociclib) days. Hospitalization and death were recorded in 54% (65% for abemaciclib) and 29% (36% for ribociclib) of cases, respectively.

#### 2.1.5 Renal toxicities

Renal alterations were more frequent with abemaciclib, with an absolute risk for any grade toxicity of 0.076 for palbociclib, 0.070 for ribociclib, and 0.261 for abemaciclib (9). Some evidence described also an increase in creatinine without change of the glomerular filtration rate (23, 24).

#### 2.1.6 Drug-drug interactions

A drug–drug interaction (DDI) is described as the ability of one drug to enhance, diminish and/or modify the action or effects of another drug when administered successively or simultaneously. DDIs are described in the Summary of Product Characteristics (SPC) of each drug, which is the reference source of information for clinicians. DDIs are a particularly important type of adverse drug event as they can alter drug effectiveness and safety; however, although not always avoidable, DDIs are predictable. The use of online tools should be viewed with great caution for several reasons. First of all, databases are largely based on the assessment of metabolic interaction, which can generate excessive warning due to their high sensitivity and overestimation of DDIs. The information retrieved from the use of on-line resources generates a clear disparity between “potential” and “clinically relevant” DDIs. Secondly, while the risk of toxicity is most frequently addressed, the impairment of treatment efficacy is not always considered. Thirdly, it is not clear if they are (i) updated, (ii) rely on good quality data, (iii) prospectively evaluated, (iv) comprehensive (i.e., if pharmacokinetic covariates are considered, including weight, fat free mass, liver/renal function, age, race, gender, clinical chemistry and hematologic values, genotype of drug metabolising enzymes and disease stage). Fourthly, online tools do not consider the therapeutic index (TI) of a drug, which is an estimate of its safety and should be included in a DDI evaluation. The TI is the ratio between toxic *vs*. clinically effective drug concentrations; a safe drug has a wide TI, while an oncology drug has typically a narrow TI. If the TI is part of a multiparametric assessment of DDIs it reduces the metabolic DDI to a subclinical entity in many cases (25).

It is, therefore, imperative to choose the most appropriate oncological drug for the patient and, after such a decision is taken, a global evaluation of the therapy by reviewing, if necessary, the non-oncological therapy that the patient is taking should be carried out. The physician should also carry out an evaluation of previous episodes of adverse reactions to drugs taken individually or in combination by the patient. It must be considered that palbociclib, ribociclib and abemaciclib are substrates of CYP3A4 and concomitant administration of CYP3A4 strong inducers or inhibitors should be avoided (25).

Finally, the online tools can provide a suggestion but cannot guide the therapy as only the technical data sheet of the drug and the professional evaluation with the help of a clinical pharmacologist specifically trained in the evaluation of DDIs are the accredited tools for this purpose.

### 2.2 Survey results

#### 2.2.1 General questions on centers characteristics, CDK4/6 inhibitors availability, and late toxicities perception

A total of 38 hospitals participated in the survey (13 centers from Italy, 6 from Belgium and Sweden, 3 from Greece and Switzerland, 2 from Austria and Germany, and 1 from Russia and the UK; one center did not indicate the country). Most centers treated more than 100 patients with metastatic breast cancer per year (50%) and from 51 to 100 patients (32%), with at least 3 doctors dedicated to breast cancer (68%, of which 18% had more than 5 dedicated doctors). Inside the hospital, most of the centers had specific gastroenterology (94.7%), pulmonology (86.8%), and cardiology (94.7%) units, whereas 7.9% of centers had no dedicated departments; permanent consultants in the above areas were available in 84% of centers.

**Figure 1:**
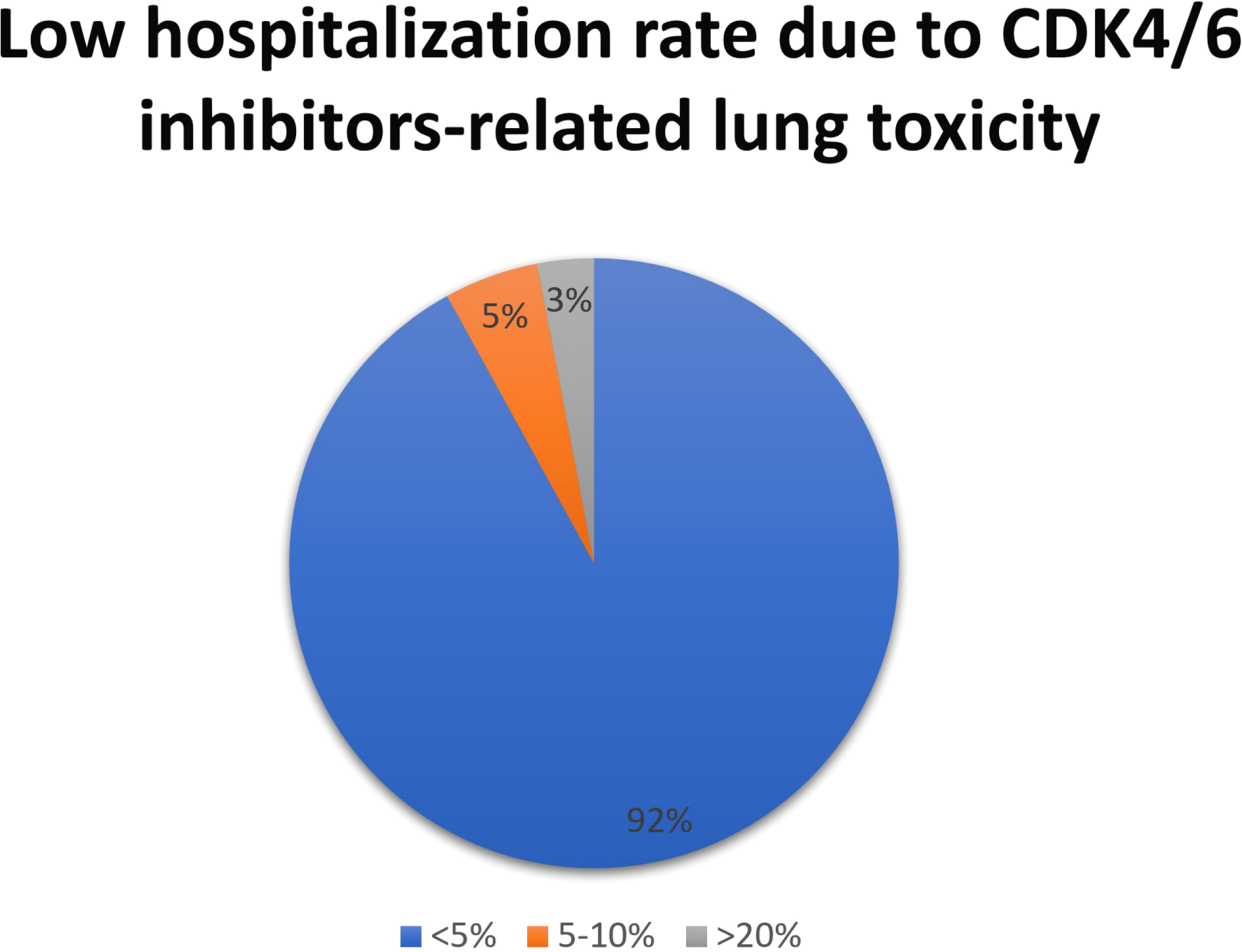
CKD4/6 inhibitors late toxicities identified after the first 24 months of treatment. ILD, interstitial lung disease.

In most countries, CDK4/6 inhibitors were available and reimbursed -only palbociclib was available but not reimbursed in one country- from 2017-2018 in most centers (65%). The topic of late toxicities was considered relevant for CDK 4/6 inhibitors by 68% of respondents to the survey, while 24% of participants were not particularly concerned about this potential issue. Hematological toxicities (39%) and combined hematological and gastro-enteric toxicities (34%) were the most common adverse reactions identified after 24 months of treatment in clinical practice; pulmonary and vascular-embolic toxicities were also reported with a lower frequency (**Figure 1**). These toxicities had an impact on patient management for 74% of respondents.

#### 2.2.2 Focus on hematological toxicity and infections

**Figure 2:**
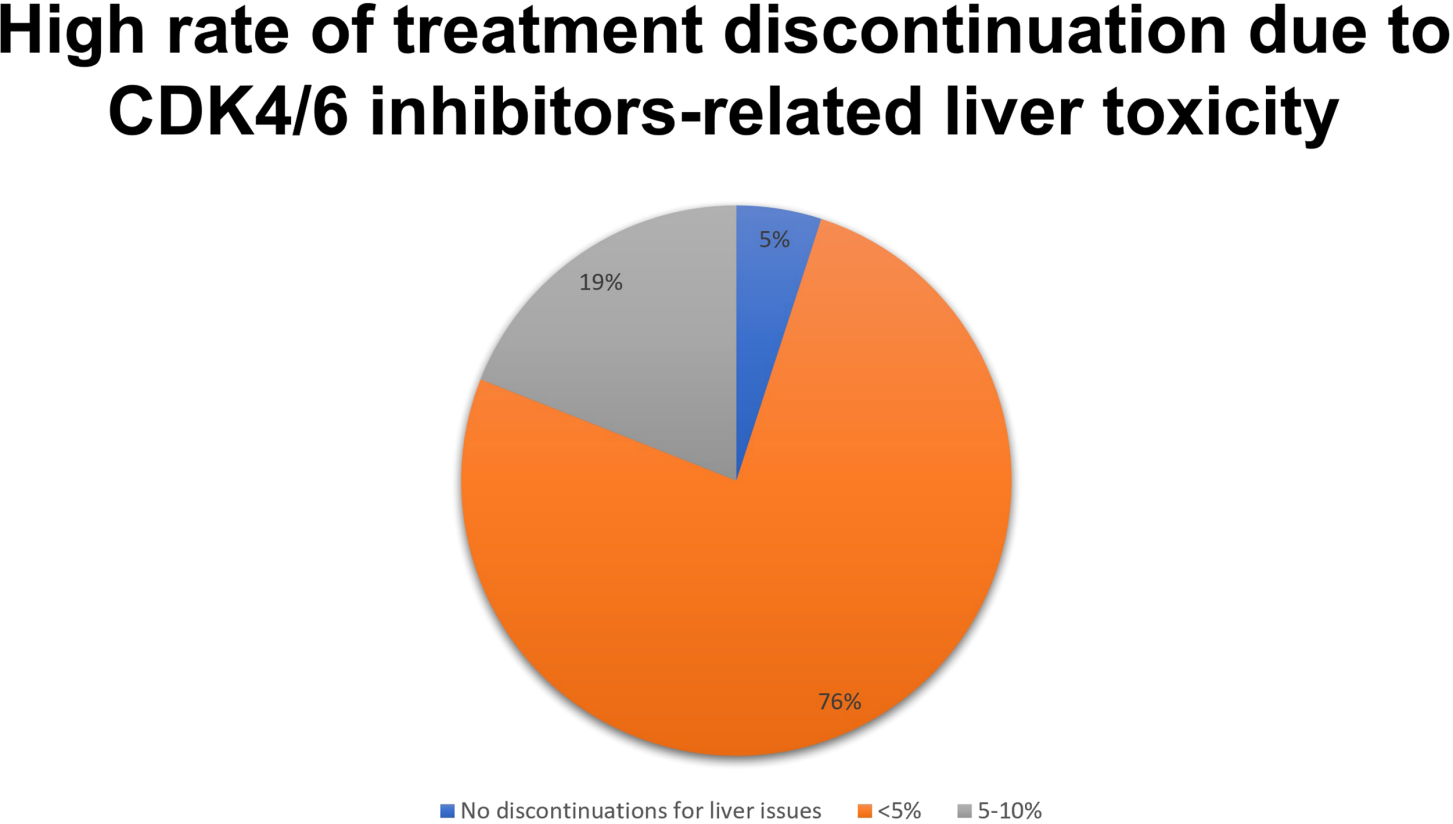
Results to the following question: Is neutropenia a major cause of CDK 4/6 inhibitors discontinuation in the clinical practice?

Hematological toxicity was considered the most common adverse reaction during the first 24 months of treatment. Neutropenia represented the most frequent hematologic side effect of CDK4/6 inhibitors treatment; however, for only 5% of respondents neutropenia was a relevant cause of treatment discontinuation for the outcome (**Figure 2**). Neutropenia of grade 3-4 represented a clinically relevant infectious risk for most respondents (95%), but it was an occasional event occurring in a minority of patients. Most of the respondents (61%) stated that they do not follow a specific protocol for the management of neutropenia but managed it on a case-by-case basis, the others had a specific protocol for the use of G-CSF and antibiotic prophylaxis.

#### 2.2.3 Focus on gastro-enteric and hepatic toxicity

Most of the respondents (92%) managed diarrhea by reducing the dose or withdrawing the CDK4/6 inhibitors -37% of respondents reduced the dose or withdrew in less than 5% of patients, 34% in 5-10%, and 21% in more than 10% of patients. Most participants (42%) perceived diarrhea as a frequent but easily manageable symptom, while for 34% of respondents diarrhea was a relevant cause of poor quality of life in treated patients, and for 8% there was a need for more effective approaches to treating this common event; for the remnants (16%), diarrhea was not a concern in their clinical practice.

To manage diarrhea, 76% of respondents administered loperamide at first loose stool, 21% prescribed loperamide if no benefit derived from diet and hydration, and 3% prescribed only diet plus hydration without drugs. The choice of loperamide is reasonable as this weak opioid agonist acts both by reducing peristaltic propulsion of the bowel and inhibiting the overall fluid secretion in the lumen. This combined mechanism of action makes loperamide a first-line option for most cases of secretive diarrhea. An additional effect on anal sphincter tone also reduces the frequently associated urgency incontinence. Other options to manage diarrhea in this context were supplements (not further specified), diosmectite, otilonium bromide, cholestyramine, tincture opii (i.e. Dropizol®), and codeine. No other relevant GI symptoms (nausea, loss of appetite, and abdominal pain and discomfort) were of concern for most respondents (63%).

**Figure 3:**
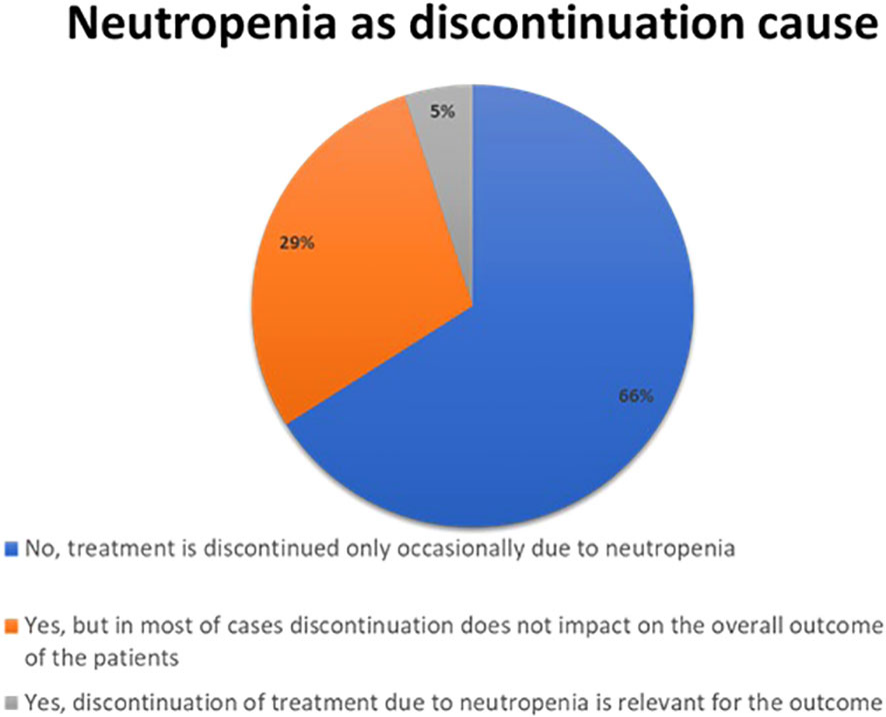
Results to the question: How many patients treated had to discontinue CKD 4/6i due to liver toxicity?

Liver toxicity of CDK4/6 inhibitors led to some degree of treatment discontinuation for the vast majority (95%) of respondents (**Figure 3**).

As the severity of liver events is greater when bilirubin elevation is associated with enzymes abnormalities and, in a setting of drug toxicity, this event is linked to a 10%-risk of mortality (so-called Hy’s law), a specific question of the survey concerned the issue with significant bilirubin elevation (bilirubin >3 mg/dl): 42% of participants reported no cases of bilirubin elevation above 3 mg/dl, 29% reported a single case per 100 treated, 21% had two cases per 100 treated, and only 8% of respondents experienced more than two cases per 100 treated (26). Overall, 58% of specialists reported at least one Hy’s law event per 100 patients treated in their clinical practice.

Most participants considered liver toxicity neither so frequent to warrant attention by researchers (37%) nor so impactful to raise concerns, regardless of its frequency (29%). However, a quarter of respondents highlighted a strong need for new knowledge about the topic and 8% of them deemed liver toxicity an event with a significant impact on the patient. The major concern about the liver toxicity of CDK4/6 inhibitors resulted in the discontinuation of treatment (45%), followed by the delay in resuming treatment itself (42%). Fewer participants pointed to the increased number of visits and examinations (8%), and liver health issues (5%). According to some respondents in the free comments section, switching from ribociclib to another CDK4/6 inhibitor was considered a successful action.

As pre-existing liver conditions may affect the on-treatment risk of developing liver toxicity, participants were asked to describe their screening practice. 47% of the oncologists used a standard liver biochemistry panel with or without virus testing, while 29% of them prescribed a more complete assessment including liver biochemistry, serology for HCV and HBV, iron overload and metabolic syndrome lab tests, and ultrasound or magnetic resonance imaging to detect steatosis. No specific liver screening was provided by 21% of respondents. Lastly, participants were asked to describe which liver screening bundle they deemed feasible in their center, choosing the highest level affordable at the respective local practice: 26% of oncologists chose the basic bundle including ALT, AST, triglycerides, bilirubin, without *ad-hoc* abdominal imaging; a similar package with the adjunct of ferritin and HBsAg was chosen by 5%, while 21% added serum protein electrophoresis and HCVAb. In 29% of cases, a configuration including all the above-mentioned exams plus an upper abdomen US or MRI was indicated. Finally, 19% of participants deemed feasible a complete bundle plus transient or shear-wave elastography. No further information on the use of fibroelastometry service with the controlled attenuation parameter (CAP) function for the quantitative assessment of steatosis was retrieved in the survey, as only 7 oncologists answered the specific question on fibroscan availability (all of them having access to this facility).

#### 2.2.4 Focus on pulmonary toxicity

**Figure 4:**
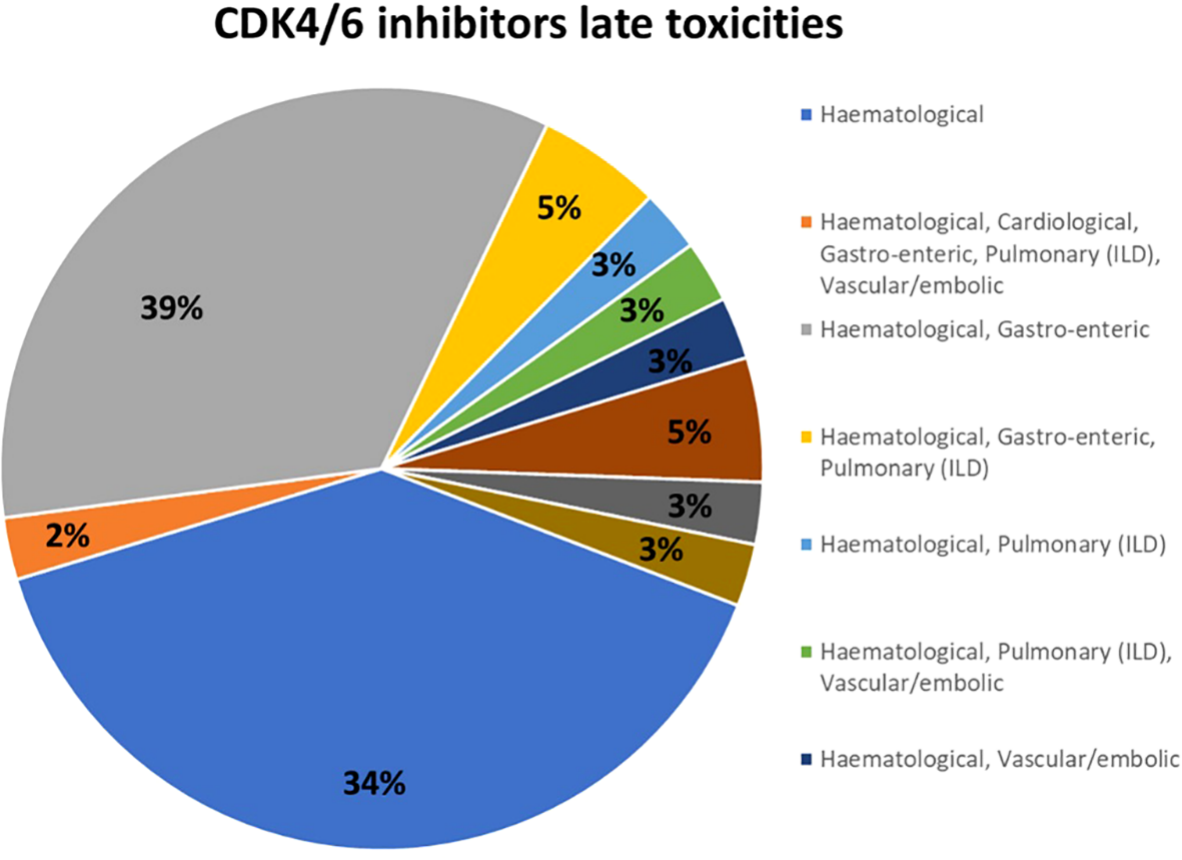
Results to the question: What is the percentage of patients treated with CKD 4/6i who developed lung toxicity requiring hospitalization?

All respondents together stated that less than 5 patients discontinued the treatment for lung toxicity. Within this subgroup, at the high-resolution computed tomography (HR-CT) of the chest, the most frequent radiological pattern was the “ground-glass” (53%), followed by both an organizing pneumonia pattern as well as the usual interstitial pneunomia pattern (5% each); 37% of respondents did not provide an adequate response (pattern not known). When lung toxicity occurs, 76% of respondents withdrew the drug and initiated high-dose steroids; in a lower number of cases, patients were managed with drug withdrawal with (13%) or without (11%) low-dose steroids. Considering the tools to follow up pulmonary toxicity, in 39% of the cases lung function tests, including carbon monoxide diffusing capacity, were utilized; in other centers, the follow-up was performed using respiratory symptoms or chest X-ray (29% each); only in a minority of cases (3%), follow-up was provided by chest ultrasound. Most respondents (92%) stated that CDK4/6 inhibitors-related lung toxicity required hospitalization in less than 5% of patients (**Figure 4**).

#### 2.2.5 Other toxicities and potential drug-drug interactions

Respondents were, then, asked about the rate of discontinuation due to persistent skin toxicity and they stated that only a minority of patients interrupted the treatment for this reason. Furthermore, according to 62% of respondents, only in a minority of patients did persistent skin toxicity had an impact on treatment adherence, while for 18% of respondents skin toxicity affected adherence, and 21% did not have an impact on this. Similarly, 61% of respondents did not think that protracted nausea could affect the quality of life and adherence to treatment. In elderly patients with more comorbidities, for 71% of respondents, there was no fear of drug interactions; they evaluated potential drug interactions and modified concomitant therapies to allow CDK4/6 inhibitors intake. Concomitant medication changes were managed autonomously (31%), with a pharmacologist consultant (24%), and with the consultant according to the associated disease (45%). All oncologists used an electronic device to evaluate drug-drug interactions, the most commonly used was the Drug Interaction Checker on the drugs.com website.

## 3 Actionable recommendation sand discussion

### 3.1 Roadmaps for the management of long-term toxicities

The attitudes of European oncologists who responded to the survey mirrored the evidence that emerged from randomized clinical trials and real-world experience reported in the literature. The following suggestions are based on the personal experience of a single expert -pulmonologist, hematologist, and gastroenterologist-, and are not the result of a consensus. During the plenary session, these suggestions were discussed with the oncologists and their feasibility in clinical practice was verified.

Among hematological toxicities, neutropenia is a common side effect of CDK4/6 inhibitors treatment (more common with palbociclib and ribociclib compared to abemaciclib), but it is often not relevant for the clinical outcomes. It spontaneously reverts with drug interruption, and it does not need to be treated with granulocyte-colony stimulating factor (G-CSF), as per prescribing information (**Box 1**). As the treatment with CDK4/6 inhibitors is not immuno-suppressive, no concerns arise in administering a vaccine in neutropenic patients, and no evidence suggests the need to delay or postpone a vaccine.

**Figure:**
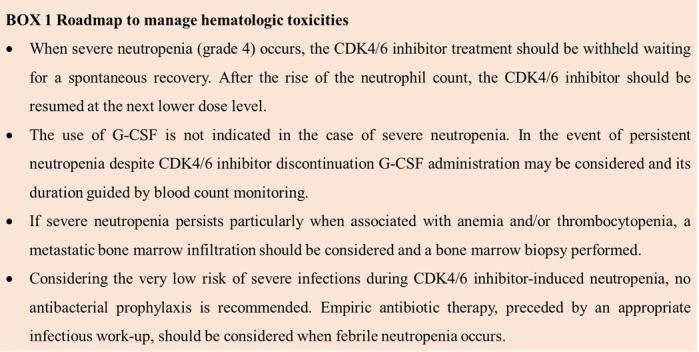

Among gastrointestinal adverse reactions, diarrhea is very frequently associated with abemaciclib-containing regimens, it is an early side effect of mild to moderate severity in most cases. Loperamide is to be given at the first sign of loose stool, starting with a low-medium dose (2 mg twice to thrice a day) and escalating if needed to a maximum of 12 mg a day (4 mg thrice a day). In case of high intestinal output (i.e. 6 or more loose stool evacuations), always associate adequate hydration with oral fluids and electrolytes. Based on clinical experience, there is a potential warning of liver toxicity when CDK4/6 inhibitors are administered. Potential liver safety issues deserve special attention. As per the prescribing information, transaminase levels should be monitored during the treatment; in case of elevation, dose modifications should be considered. Steatosis and hypertriglyceridemia are likely associated with an augmented risk of CDK4/6-induced liver injury. As these features are usually encompassed in the metabolic syndrome, dedicated counseling, including diet and physical training recommendations, with or without drug intervention, may totally or partially reverse predisposing conditions thus reducing the risk of overall drug toxicity (**Box 2**).

**Figure:**
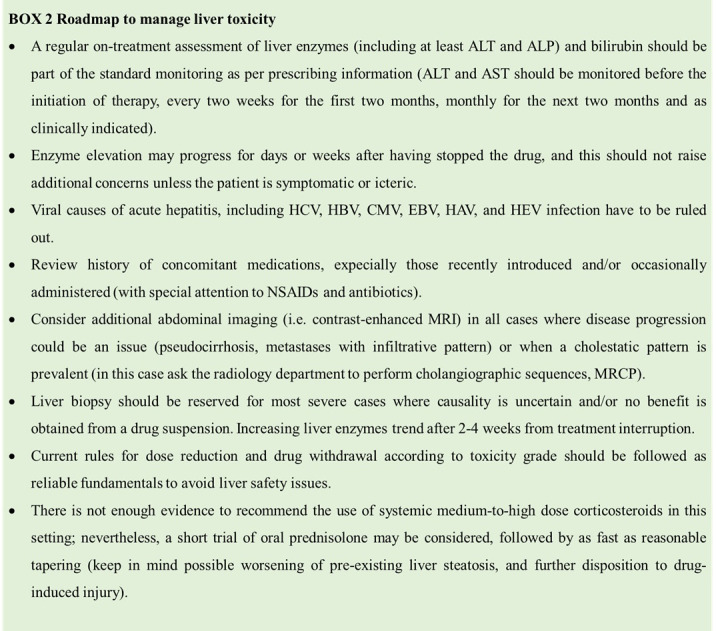

Pulmonary toxicities appear with specific symptoms, including dyspnea, dry cough, low-grade fever, and chest pain; early clinical signs are usually a dry cough and progressive dyspnea. As most cases of ILD secondary to CDK4/6 inhibitors occur during the earlier months of treatment, clinicians should pay particular attention to the start of treatment. Patient management is currently guided by clinical experience. However, per prescribing information, depending on ILD/pneumonia severity, the dosage of CDK4/6 inhibitors may need to be adjusted or permanently discontinued (grade 3-4 ILD/pneumonia) (27–29). The safety of CDK4/6 inhibitors rechallenge after resolution of grade 3 pneumonitis is debatable but is not recommended in most cases. Decisions to re-treat should be systematically discussed in the context of a multidisciplinary team, calibrated against the risk: benefit ratio for each patient. CDK4/6 inhibitors therapy should be permanently discontinued following grade 4 pneumonitis (**Box 3**).

**Figure:**
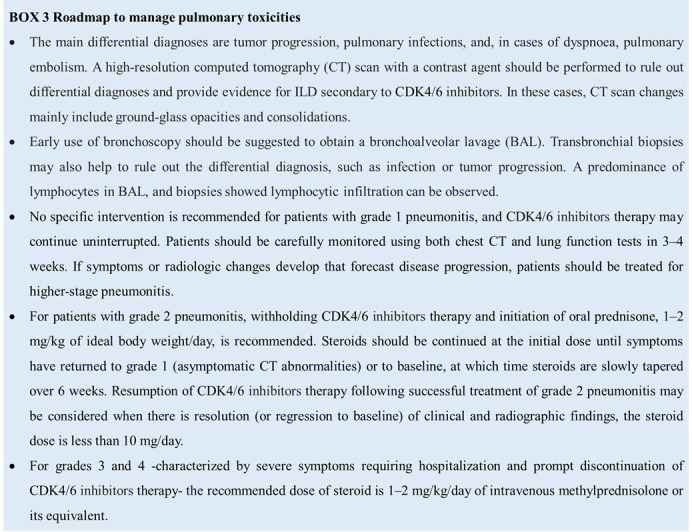

Finally, the panel acknowledged that, among non-cancer treatments, common co-medications involve antidepressants (mainly serotonin uptake inhibitors), anxiolytics (benzodiazepines), lipid-lowering drugs (statins), beta-blockers, and proton pump inhibitors. Based on the respective SPCs, the main enzyme involved in the metabolism of antidepressant is CYP2D6, while the other drugs are metabolized by CYP3A4, although multiple pathways may be involved (i.e., bisoprolol is metabolized by CYP3A4 and to a lesser extent by CYP2D6, most statins are metabolized by both CYP3A4 and 2C9, bromazepam is metabolized by CYP2D6 and 1A2 and diazepam is metabolized by CYP3A4 and 2C19) or not involved at all (i.e., atenolol and lorazepam are not metabolized by CYPs). A CYP-independent effect is observed with proton pump inhibitors, which may affect the bioavailability of selected CDK4/6i by impairing their solubility in the gastrointestinal tract. Therefore, due to the complexity of metabolic pathways and the different TIs of many drugs frequently taken in multiple combinations (i.e., based on the severity and frequency of adverse events listed in the SPCs most of these drugs have high TI, while only statins have intermediate TI), only a professional advice allows a safe treatment of patients.

## 4 Conclusion

The use of CDK4/6 inhibitors has changed the management of patients with metastatic breast cancer and improved the outcomes with a manageable safety profile.

Recently, Abemaciclib (30) and Ribociclib (31) demonstrated to significantly improve the iDFS in intermediate and high risk populations of early HR+/HER2- breast cancer patients. These results, so important in the context of a curative setting, should be paired with an optimized management of side effects: despite the lower dose of Ribociclib (400 mg/day) used in the NATALEE trial, incidence of Grade 3-4 neutropenia was 43.8% and 25.4% for any grade liver toxicity. A careful attention in monitoring longterm adverse events and a roadmap to guide physicians all over the world in the management of these toxicities is strongly recommended.

Specific toxicity profiles with diarrhea for abemaciclib and neutropenia for palbociclib and ribociclib emerged from the clinical trials and everyday practice, while special attention should be reserved for hepatic and pulmonary toxicities. Careful monitoring of liver function and the identification of signs and symptoms of ILD could allow to avoid worse injuries and preserve organ functionality. Further studies in the real world should better clarify the safety profile of these agents.

## Data availability statement

The raw data supporting the conclusions of this article will be made available by the authors, without undue reservation.

## Author contributions

All Authors fulfil ICMJE criteria for authorship: they substantially contributed to the design of the work and data acquisition; they revised the manuscript critically for important intellectual content and provided approval for publication of the content. They agree to be accountable for all aspects of the work in ensuring that questions related to the accuracy or integrity of any part of the work are appropriately investigated and resolved. All authors contributed to the article and approved the submitted version.
