# Supplementary material for: Late onset toxicities associated with the use of CDK 4/6 inhibitors in hormone receptor positive (HR+), human epidermal growth factor receptor-2 negative (HER2-) metastatic breast cancer patients: a multidisciplinary, pan-EU position paper regarding their optimal management. The GIOCONDA project
Source: Front Oncol. 2023 Oct 26;13:1247270. doi: 10.3389/fonc.2023.1247270 (PMC10639170; doi:10.3389/fonc.2023.1247270)
Supplement: Supplementary file 1 [file DataSheet_1.docx]

**References Supplementary Table 2**

19. Fuentes-Antras J, de Luna A, Lopez de Sa A, Ocana A, Garcia-Saenz JA and Moreno F. Safety and efficacy of cyclin-dependent kinase inhibitor rechallenge following ribociclib-induced limiting hypertransaminasemia. Breast (2020) 54:160-63. doi: 10.1016/j.breast.2020.10.007

32. Elder C, Ohana Z and Younas W. Fulvestrant- and Palbociclib-Induced Hepatotoxicity in a Patient with Breast Cancer: A Case Report. J Hematol Oncol Pharm. (2020) 10:235-40.

18. Finnsdottir S, Sverrisdottir A and Bjornsson ES. Hepatotoxicity associated with ribociclib among breast cancer patients. Acta Oncol (2021) 60:195-98. doi: 10.1080/0284186X.2020.1853228

33. Schlotman A, Stater A, Schuler K, Heideman J and Abramson V. Grade 3 Hepatotoxicity following Fulvestrant, Palbociclib, and Erdafitinib Therapy in a Patient with ER-Positive/PR-Negative/HER2-Negative Metastatic Breast Cancer: A Case Report. Case Rep Oncol (2020) 13:304-08. doi: 10.1159/000506442

34. Topcu A, Yasin AI, Shbair AT, Besiroglu M, Simsek M, Sucuoglu Z, et al. A case report of fulminant hepatitis due to ribociclib with confirmed by liver biopsy in breast cancer. J Oncol Pharm Pract (2022) 28:242-46. doi: 10.1177/10781552211027931

35. Hyppolite JJ and Hilzenrat N. Palbociclib-induced severe hepatitis: A case study and literature review. Can Liver J (2021) 4:433-37. doi: 10.3138/canlivj-2021-0015

36. Meynard L and Grellety T. CDK 4/6 inhibitor successful rechallenge after limiting hepatic toxicity. Breast J (2020) 26:255-57. doi: 10.1111/tbj.13532

14. Rugo HS, Huober J, Garcia-Saenz JA, Masuda N, Sohn JH, Andre VAM, et al. Management of Abemaciclib-Associated Adverse Events in Patients with Hormone Receptor-Positive, Human Epidermal Growth Factor Receptor 2-Negative Advanced Breast Cancer: Safety Analysis of MONARCH 2 and MONARCH 3. Oncologist (2021) 26:e53-e65. doi: 10.1002/onco.13531

**References Supplementary Table 3**

37. Algwaiz G, Badran AA, Elshenawy MA and Al-Tweigeri T. Ribociclib-Induced Pneumonitis: A Case Report. Breast Care (Basel) (2021) 16:307-11. doi: 10.1159/000507647

38. Felip E, Llobera L, Perez-Mana C, Quintela D, Guasch I, Margeli M, et al. New Drugs, Old Toxicities: Pneumonitis Related to Palbociclib - A Case Report. Breast Care (Basel) (2020) 15:548-52. doi: 10.1159/000504618

39. Mathew N, Joel A, Andrews AG, John AO and Singh A. CDK 4/6 inhibitor induced lung injury: a case report and review of literature. Ecancermedicalscience (2021) 15:1245. doi: 10.3332/ecancer.2021.1245

40. Mitarai Y, Tsubata Y, Hyakudomi M and Isobe T. Drug-Induced Eosinophilic Pneumonia as an Adverse Event of Abemaciclib. Cureus (2022) 14:e21741. doi: 10.7759/cureus.21741

22. Raschi E, Fusaroli M, Ardizzoni A, Poluzzi E and De Ponti F. Cyclin-dependent kinase 4/6 inhibitors and interstitial lung disease in the FDA adverse event reporting system: a pharmacovigilance assessment. Breast Cancer Res Treat (2021) 186:219-27. doi: 10.1007/s10549-020-06001-w
