# Supplementary material for: Late onset toxicities associated with the use of CDK 4/6 inhibitors in hormone receptor positive (HR+), human epidermal growth factor receptor-2 negative (HER2-) metastatic breast cancer patients: a multidisciplinary, pan-EU position paper regarding their optimal management. The GIOCONDA project
Source: Front Oncol. 2023 Oct 26;13:1247270. doi: 10.3389/fonc.2023.1247270 (PMC10639170; doi:10.3389/fonc.2023.1247270)
Supplement: Supplementary file 2 [file Table_1.docx]

**Supplementary materials**

**Supplementary Table 1 Survey**

| Question | Answer (%) |
| --- | --- |
| How many metastatic breast cancer patients do you treat per year?   1. 10-50 2. 51-100 3. >100 | 19  7  12 |
| How many doctors in your team are dedicated to breast cancer?   1. <3 2. 3-5 3. > 5 | 12  7  19 |
| Does your hospital have the following facilities inside?   1. Gastroenterology Unit 2. Pulmonology Unit 3. Cardiology Unit 4. There are no such departments | 94.7  86.8  94.7  7.9 |
| Do you have permanent consultants in the above-mentioned areas?   1. Yes 2. No | 84  16 |
| Are CDK 4/6 inhibitors available in your Country:   1. Available and reimbursed 2. Available but not reimbursed 3. Available only on an out-of-pocket regimen | 97  3  0 |
| Which of the following CDK 4/6 inhibitors are available, even if not reimbursed?   1. Palbociclib 2. Ribociclib 3. Abemaciclib   Since what year are they available (for the first one)? | 97 all  3 only palbociclib  2014 3%  2015 5%  2016 5%  2017 31%  2018 34%  2019 3%  Unknown 11% |
| Do you think the topic of late toxicities is relevant for CDK 4/6 inhibitors?   1. Yes 2. No 3. Don’t know | 68  24  8 |
| In your clinical practice, which are the most common toxicities you identified after the first 24 months of treatment for concerned patients? (multiple choice allowed)   1. Hematological 2. Cardio-vascular 3. Gastro-enteric 4. Pulmonary (ILD) 5. Vascular/embolic | 87  2  42  21  5 |
| In your opinion, do these toxicities have an impact on patient management?   1. Yes 2. No | 74  26 |
| Is neutropenia a major cause of CDK 4/6 inhibitors discontinuation in clinical practice?   1. No, treatment is discontinued only occasionally due to neutropenia 2. Yes, but in most cases, discontinuation does not impact the overall outcome of the patients 3. Yes, discontinuation of treatment due to neutropenia is relevant for the outcome | 66  29  5 |
| Does grade 3/4 neutropenia represent a clinically relevant infectious risk?   1. Yes, but in a minority of patients (occasional events) 2. Yes, a significant proportion of patients 3. Don’t know | 95  3  2 |
| Is a defined neutropenia management protocol applied in your center?   1. No, neutropenia is managed on a case-by-case basis 2. Yes, in my center we defined the indications for the use of G-CSF and/or antibiotic prophylaxis | 61  39 |
| Concerning digestive/GI symptoms, did you experience cases needing dose reduction or treatment withdrawal?   1. Yes, <5% 2. Yes, 5-10% 3. Yes, >10% 4. None | 37  34  21  8 |
| Which sentence best apply to the symptom of diarrhea?   1. It is a relevant cause of poor quality of life in treated patients 2. It is frequent but manageable 3. More effective approaches than those available are lacking 4. It is not a concern in my practice | 34  42  8  16 |
| How do you manage diarrhea? (multiple choice allowed)   1. Diet only plus hydration 2. Loperamide at first loose stool 3. Loperamide if no benefit from diet or selected cases 4. Other drugs and/or actions, please specify \|____________________\| | 3  76  21  0 |
| Do you have any relevant concerns about other GI symptoms (nausea, loss of appetite, abdominal discomfort)?   1. Yes, please specify symptom \|______________\| and which CKD 4/6i causes it \|____________\| 2. No | 37  63 |
| In your experience, how many patients treated have to discontinue CKD 4/6i due to liver toxicity?   1. <5% 2. 5-10% 3. >10% 4. No discontinuation for liver issues | 76  19  0  5 |
| Did you experience any issues of significant bilirubin elevation (bilirubin > 3 mg/dl)?   1. No 2. A single case per 100 treated 3. 2 cases per 100 treated 4. > 2 cases per 100 treated | 42  29  21  8 |
| Which sentence best apply to the liver adverse effects (grade 3-4) in your practice?   1. It is relatively frequent with a significant impact on the affected patients 2. It is relatively frequent but the overall impact is limited 3. It is not so frequent to warrant attention by researchers 4. There is a strong need for new knowledge about the topic regardless of the incidence | 8  29  37  26 |
| A major concern about liver toxicity by CDK4/6i:   1. Discontinuation of CDKi 2. Delay in resuming treatment (with any regimen) 3. Liver health issues 4. Increased number of exams/visits | 45  42  5  8 |
| Pre-existing liver conditions may affect the on-treatment risk of developing liver toxicity, please choose the option that best describes your current practice.   1. CKD 4/6i candidates are regularly screened for HCV, HBV, iron overload, steatosis by US/MRI, and metabolic syndrome features. 2. US/MRI only (also outside neoplastic staging) 3. Only liver biochemistry with or w/o virus screening 4. No specific liver screening | 29  3  47  21 |
| Which “liver screening bundle” do you deem feasible to be performed in your Centre (please choose the highest level you can afford at your local practice)?   1. ALT, ALP, triglycerides, bilirubin, plus abdominal imaging already available 2. ALT, ALP, triglycerides, bilirubin, ferritin, HBsAg, plus abdominal imaging already available. 3. ALT, ALP, triglycerides, bilirubin, ferritin, HBsAg, anti-HCV, protein electrophoresis, plus abdominal imaging already available. 4. All those mentioned in item c, plus upper abdomen US or MRI for steatosis detection. 5. All those mentioned in item c, plus transient elastography (Fibroscan®) or US-based shear wave elastography (pSWE) for fibrosis/steatosis detection. | 26  5  21  29  19 |
| How many patients treated with CKD 4/6i discontinued the drug because of lung toxicity?   1. <5% 2. >5% | 100  0 |
| What is the main chest HRCT pattern observed in the case of pulmonary toxicity secondary to CDK 4/6i?   1. Organizing pneumonia pattern 2. Ground glass opacity pattern 3. Honey combing pattern 4. I don’t know | 5  53  5  37 |
| What is the best treatment when lung toxicity secondary to CDK 4/6i occurs?   1. Drug withdrawal together with high-dose steroids 2. Drug withdrawal together with low-dose steroids 3. Drug withdrawal | 76  13  11 |
| What is the best tool to follow up lung toxicity secondary to CDK 4/6i?   1. Lung function tests, including carbon monoxide diffusing capacity 2. Respiratory symptoms 3. Chest ultrasounds 4. Chest x-ray | 39  29  3  29 |
| What is the percentage of patients treated with CKD 4/6i who developed lung toxicity requiring hospitalization?   1. <5% 2. 5-10% 3. >10% | 92  5  3 |
| Have you ever experienced treatment withdrawal or dose reduction due to G3/4 neutropenia after 2 years of treatment?   1. Yes 2. Yes in a minority of patients 3. No | 24  16  60 |
| Have you ever experienced G2 persistent skin toxicity for which you had to discontinue treatment? and in what percentage?   1. Yes, often 2. Yes, only a minority of patients 3. No | 0  100  0 |
| In your opinion, could persistent skin toxicity impact adherence to treatment?   1. Yes 2. Yes, only a minority of patients 3. No | 18  61  21 |
| In your clinical experience, how much does protracted nausea impact the quality of life and adherence to therapy of your patients?   1. It can impact a lot 2. I don’t think it is important | 39  61 |
| Are there any elderly patients with more comorbidities to whom you think not to prescribe CDK4/6i for fear of drug interactions?   1. Yes 2. No, I usually evaluate if there are interactions and modify the treatment to allow the intake of CDK4/6i | 29  71 |
| Do you base yourself on the number of drugs taken or on the type?   1. Number of drugs for any other disease 2. Type of drug | 18  82 |
| In your management, do you evaluate interactions with CDK4/6i and change medication for the associated disease or do you have a pharmacologist consultant?   1. No, I decide by myself and with my patient 2. I contact the consultant according to the associated disease 3. I have a pharmacologist consultant | 31  24  45 |
| Do you use any electronic device to evaluate drug-drug interactions?   1. WebMD interaction checker 2. Drugs.com (Drug Interaction checker) 3. Drugs.com (Drug Interaction checker), Others 4. Drugs.com (Drug Interaction checker), WebMD interaction checker 5. Drugs.com (Drug Interaction checker), WebMD interaction checker, Others 6. Others | 13  34  3  13  5  32 |

**Supplementary Table 2 Case Reports on hepatic toxicity**

| **REF** | **Study design** | **Population** | **CDK4/6 inhibitor** | **Adverse reactions** | **Action and comments** |
| --- | --- | --- | --- | --- | --- |
| [19] | Retrospective study | 64 HR+/HER2- MBC patients (1 with liver metastasis, 1 with brain metastasis) | Ribociclib (median 4.5 cycles), median follow-up 19.5 months | Hypertransaminasemia of any grade occurred in 14 patients (21.9%), of which grade 3 occurred in 7 patients (10.9%). | Ribociclib was interrupted and reintroduced with dose reductions or discontinued. |
| [32] | Case report | 58-year-old postmenopausal woman | Palbociclib + fulvestrant | Elevation of liver transaminases (peak ALT >700 and AST 421). Total bilirubin remained stable. | Re-introduction of fulvestrant monotherapy with normal liver function. Palbociclib (100 mg) was re-introduced 3 months later. The elevation in liver function tests occurred within 1 week of restoring palbociclib therapy. |
| [18] | Case report | First case: a 54-year-old woman with disseminated bone metastases.  Second case: 66-year-old woman | First case: Ribociclib for a total of 21 d of treatment. Second case: Ribociclib 3 cycles | First case: after the first cycle of ribociclib, occurred pruritus and ALT elevation. Liver enzymes continued rising despite discontinuation of the drug with ALT > 1000 (U/L), concomitant jaundice, and coagulopathy (INR 2.1) but no encephalopathy. Ultrasound of the liver did not demonstrate any liver metastases and a CT scan of the liver was normal. Second case: after 3 cycles, liver enzymes were elevated and continued to rise after drug discontinuation (peak after 5 weeks 1112 U/L). Nonspecific symptoms of nausea and fatigue but no pruritus, jaundice, or encephalopathy (INR 1.3) were reported. | First case: As liver tests remained elevated despite cessation of therapy, treatment with prednisolone (40 mg) was initiated. Liver enzymes normalized a month later with prednisolone doses tapered off.  Second case: prednisolone (40 mg) was initiated. Liver enzymes normalized after five weeks of corticosteroid treatment in doses that were tapered off. |
| [33] | Case report | A 49-year-old woman with metastasis to bone and liver | Palbociclib, fulvestrant, and erdafitinib in a clinical trial | During the first week of the first cycle, the patient reported fatigue and nausea, followed by watery pale diarrhea, and crampy abdominal pain in the right upper quadrant and mid-epigastric areas. Then, despite stopping oral medications, the patient had markedly elevated liver enzymes. The patient remained asymptomatic. | Oral drug interruption.  This patient reported symptoms onset shortly after the fulvestrant injection and her symptoms improved over the next week despite continuing to take erdafitinib and palbociclib. She had been treated previously with a 5-month course of palbociclib with no adverse reaction or liver injury, thus making palbociclib unlikely to be the culprit, while the culprit was likely fulvestrant |
| [34] | Case report | A 54-year-old woman with multiple lung and bone metastases. | Ribociclib + fulvestrant | After 8 weeks, increase in the serum levels of liver transaminases, initially grade 1 for ALT and ASP, then rising rapidly to grade 4. Grade 3 bilirubin elevation was detected. | Ribociclib was permanently discontinued and 1 mg/kg prednisolone therapy was initiated. Liver enzymes and bilirubin rapidly decreased and all laboratory values returned to normal within 3 weeks; the patient completely recovered clinically. A liver biopsy was compatible with drug-induced acute fulminant toxic hepatitis |
| [35] | Case report | A 61-year-old female with lymph node recurrence | Palbociclib + letrozole | After 2 weeks, liver function tests started to rise (ALT 615 IU/mL and AST 417 IU/mL and after 3 days, ALT 1713 IU/mL and AST 1052 IU/mL). No nausea, vomiting, or abdominal pain. | Palbociclib was discontinued. The patient was treated with IV fluids and IV N-acetyl cysteine (NAC) 300 mg/kg for 21 hours.  Treating patients with NAC for non-acetaminophen-induced DILI is, however, controversial. |
| [36] | Case report | A 59‐year‐old woman with diffuse bone metastasis | Ribociclib + letrozole | After 16 weeks, transaminases initially increased to grade 1-2, then rose rapidly to grade 3 | Ribociclib was discontinued; once the liver function normalized, palbociclib was introduced at 75 mg per day, and then increased to 100 mg per day. One year on, the patient remains free of any clinical or biological toxicities. |
| [14] | RCT | 768 patients included in MONARCH 2 and 3 trials | Abemaciclib + fulvestrant or a nonsteroidal aromatase inhibitor | In the abemaciclib arms, any-grade increased ALT occurred in 13%–17%; any-grade increased AST occurred in 12%–17% of patients. The median time to onset for grade ≥3 increased ALT in the abemaciclib arms was approximately 60 days in both studies. | Dose reduction or discontinuation due to increased ALT or AST was infrequent (<1%). Effects were reversible in both studies, as demonstrated by the short duration of grade ≥3 increased ALT and AST, with a median time to resolution (for all patients regardless of dose adjustments) from the onset of approximately 2 weeks. |

**Supplementary Table 3 Case reports on pulmonary toxicity**

| **REF** | **Study design** | **Population** | **CDK4/6 inhibitor** | **Adverse reactions** | **Action and comments** |
| --- | --- | --- | --- | --- | --- |
| [37] | Case report | 46-year-old premenopausal female with locally advanced breast cancer and bone metastasis | Goserelin (3.6 mg s.c.), fulvestrant (500 mg i.m. on days 1 and 14 of the first 28-day cycle, then every 28 days), and ribociclib (600 mg daily for 21 days and 1 week off). | After 4 weeks, hospitalization for grade 4 mucositis, neutropenia, and thrombocytopenia. On admission, ribociclib was withheld until complete recovery of mucositis and hematopoietic lineages. However, there was no significant improvement in her neurological deficits, which required approximately 8 weeks. She was then resumed on the same regimen, with a dose reduction of ribociclib to 200 mg daily. While planning discharge, the patient experienced acute chest pain and shortness of breath. A CT scan with contrast was performed showing a subsegmental pulmonary embolism with bilateral ground-glass opacity more pronounced on the right side. | Ribociclib was stopped along with starting methylprednisolone (1.5 mg/kg/day), with noticeable clinical improvement within 1 week. Six weeks later, the patient showed complete recovery, clinically and radiologically |
| [38] | Case report | 57-year-old with bone metastasis; ex-smoker with no prior history of pulmonary disease | Palbociclib 125 mg/day in 4-week cycles (3 weeks on, 1 week off) plus letrozole 2.5 mg/day continuously and zoledronic acid 4 mg/every 28 days, with no other concomitant treatments. | After 3 months, the patient experienced progressive shortness of breath accompanied by a nonproductive cough and fever. She underwent a positron emission tomography-CT with a bilateral inflammatory pattern and a bronchoscopy where the bronchoalveolar lavage (BAL) showed lymphocytosis (55%) with a CD4/CD8 ratio of 0.18. | Empirical antibiotic therapy with ceftriaxone and levofloxacin was initiated without clinical improvement. Blood culture and urine antigen test of L. pneumophila and S. pneumonia were negative. Palbociclib was stopped, and prednisolone (2 mg/kg/day) was initiated, showing clinical improvement within 72h. Six weeks later, the patient presented a normal clinical exploration without any symptomatology. |
| [39] | Case report | 67-year-old post-menopausal woman with bone metastasis | Zoledronic acid and palbociclib with letrozole | After 4 months, the patient developed a dry non-productive cough and worsening breathlessness; CT showed multiple new patchy and confluent ground glass opacities in bilateral lung fields, suggestive of acute interstitial lung disease (ILD). | IV methylprednisolone with an empirical diagnosis of drug-induced pneumonitis. Palbociclib was discontinued. |
| [40] | Case report | A 45-year-old woman with lung and liver metastases | Abemaciclib + fulvestrant | After 2 weeks, the patient developed a mild cough, and peripheral blood eosinophil increased to 13.9%. A frosted glass shadow appeared in the lower lobe of the patient’s right lung on chest CT. These observations were suggestive of abemaciclib-induced eosinophilic pneumonia | Administration of steroids (prednisolone 30 mg/day) was started. Eosinophilic pneumonia has not recurred after initiating steroids, and, currently, resumption of treatment for breast cancer is being considered without abemaciclib. |
| [22] | Observational, retrospective pharmacovigilance analysis of the FAERS database | 161 patients who experienced ILD under treatment with CDK4/6 inhibitors. | CDK4/6 inhibitors | ILD represented 2.1% of total reports recorded for abemaciclib, as compared to 0.3% for palbociclib and ribociclib.  Most ILD reports occurred in subjects aged >65 years (72%); ILD was reported to occur at therapeutic doses (250, 125, and 600 mg daily for abemaciclib, palbociclib, and ribociclib, respectively). Hospitalization and death were recorded in 54% (65% for abemaciclib) and 29% (36% for ribociclib) of cases, respectively. Causality assessment was highly probable in 35% of ILD cases (55% for abemaciclib) and probable in 61% (68% for palbociclib). | There is a consistently increased reporting of ILD with CDK4/6 inhibitors as a class, which was consistent across the various sensitivity analyses accounting for major biases and confounders, especially for abemaciclib. |
